# Indolin-2-one compounds targeting thioredoxin reductase as potential anticancer drug leads

**DOI:** 10.18632/oncotarget.9579

**Published:** 2016-05-24

**Authors:** Kamila K. Kaminska, Helene C. Bertrand, Hisashi Tajima, William C. Stafford, Qing Cheng, Wan Chen, Geoffrey Wells, Elias S.J. Arner, Eng-Hui Chew

**Affiliations:** ^1^ Department of Pharmacy, Faculty of Science, National University of Singapore, S117543, Republic of Singapore; ^2^ UCL School of Pharmacy, University College London, London WC1N 1AX, United Kingdom; ^3^ Division of Biochemistry, Department of Medical Biochemistry and Biophysics, Karolinska Institutet, SE-171 77, Stockholm, Sweden; ^4^ Current address: École Normale Supérieure, PSL Research University, Département de Chimie, Sorbonne Universités, UPMC Univ Paris 06, CNRS UMR 7203 LBM, 75005 Paris, France

**Keywords:** anticancer, indolin-2-one, supercinnamaldehyde, selenocysteine, thioredoxin reductase

## Abstract

Several compounds bearing the indolinone chemical scaffold are known to possess anticancer properties. For example, the tyrosine kinase inhibitor sunitinib is an arylideneindolin-2-one compound. The chemical versatility associated with structural modifications of indolinone compounds underlies the potential to discover additional derivatives possessing anticancer properties. Previously synthesized 3-(2-oxoethylidene)indolin-2-one compounds, also known as supercinnamaldehyde (SCA) compounds in reference to the parent compound 1 [1-methyl-3(2-oxopropylidene)indolin-2-one], bear a nitrogen-linked α,β-unsaturated carbonyl (Michael acceptor) moiety. Here we found that analogs bearing *N*-substituents, in particular compound 4 and 5 carrying an *N*-butyl and *N*-benzyl substituent, respectively, were strongly cytotoxic towards human HCT 116 colorectal and MCF-7 breast carcinoma cells. These compounds also displayed strong thioredoxin reductase (TrxR) inhibitory activity that was likely attributed to the electrophilicity of the Michael acceptor moiety. Their selectivity towards cellular TrxR inhibition over related antioxidant enzymes glutathione reductase (GR), thioredoxin (Trx) and glutathione peroxidase (GPx) was mediated through targeting of the selenocysteine (Sec) residue in the highly accessible C-terminal active site of TrxR. TrxR inhibition mediated by indolin-2-one compounds led to cellular Trx oxidation, increased oxidative stress and activation of apoptosis signal-regulating kinase 1 (ASK1). These events also led to activation of p38 and JNK mitogen-activated protein kinase (MAPK) signaling pathways, and cell death with apoptotic features of PARP cleavage and caspase 3 activation. In conclusion, these results suggest that indolin-2-one-based compounds specifically targeting TrxR may serve as novel drug leads for anticancer therapy.

## INTRODUCTION

Due to aberrant metabolic events and abnormal proliferative growth phenotypes, reactive oxygen species (ROS) levels are commonly elevated in cancer cells [references cited in 1, 2]. To cope with an increase in intrinsic oxidative stress, cancer cells augment their antioxidant capacity through upregulation of antioxidant enzymes. In doing so, cancer cells become more dependent on the elevated antioxidant capacity for growth and survival, which makes them susceptible when oxidative stress is further elevated through production of more ROS and/or inhibition of elevated antioxidant enzymes. The selective targeting of cancer cells on the basis of different redox states between normal and tumor cells is therefore a feasible strategy [1, 3–6], particularly when it involves targeting a redox adaptive mechanism that confers drug resistance [2]. In this context, inhibition of the thioredoxin (Trx) system has emerged as a promising redox-modulating strategy for anticancer therapy. The Trx system, together with the glutathione system, forms the two main cellular thiol antioxidant systems that can be targeted for anticancer therapy [7, 8]. The Trx system comprises Trx, thioredoxin reductase (TrxR) and NADPH, where Trx executes antioxidant roles directly by reducing protein disulfides, as well as indirectly by donating reducing equivalents to peroxide scavenging enzymes such as peroxiredoxins [9] or reductive repair enzymes such as methionine sulfoxide reductases [10] or sulfiredoxin [11]. In addition, through Trx's involvement in DNA synthesis, its role in the enzymatic regulation of several redox-sensitive transcription factors, and its direct binding to and inhibition of apoptosis signal regulating kinase 1 (ASK1), the Trx system is highly important for multiple cellular aspects involving cell survival and proliferation [12, 13].

In carcinogenesis, several lines of evidence have suggested that the Trx system can support tumor growth and progression. Firstly, TrxR, the enzyme recycling oxidized Trx to its reduced form, has been found to be essential for tumorigenesis in mouse xenograft models [14]. Secondly, Trx and TrxR are found to be overexpressed in a number of human cancer cell lines and primary tumors, and their overexpression is associated with tumor aggressiveness, cancer drug resistance, and poor prognosis [15–25]. TrxR is furthermore a promising molecular target for anticancer therapy given that its inhibition may lead to directly muted activities of TrxR as well as downstream inhibition of components in the Trx pathway. TrxR is an attractive target in view of its druggability by virtue of the presence of an easily accessible selenocysteine (Sec) residue located in the C-terminal active center of the enzyme [26]. The selenol group within a Sec residue is fully ionized at physiological pH, making the nucleophilic selenolate anion available for targeting by electrophilic compounds. Among classes of compounds currently known to inhibit TrxR, several are drugs used in anticancer therapy and of these many contain α,β-unsaturated carbonyl (Michael acceptor) moieties that are perceived to target nucleophilic Sec and Cys residues in the C- and/or N-terminal active site of mammalian TrxR through Michael addition reactions [27–29].

Compounds bearing the indolin-2-one chemical scaffold have been the focus of several biological studies [30–34]. In the context of discovering anticancer agents, the tyrosine kinase inhibitor sunitinib indicated for the treatment of renal cell and gastrointestinal stromal tumors [35, 36] is an arylideneindolin-2-one compound. 3-Benzylidene-indolin-2-ones are also promising compounds for the treatment of hepatocellular carcinoma and have been found to inhibit multiple tyrosine kinases [37]. In another study that investigated the NAD(P)H:quinone oxidoreductase 1 (NQO1) inducing activity of a series of 3-benzylidene-indolin-2-ones, most of the test compounds were found to cause induction of NQO1, a cytoprotective enzyme whose upregulation is usually associated with chemopreventive potential [38]. Conversely, fewer compounds registered strong anti-proliferative activities [38]. Derivatives carrying N-substituents of indolinone compounds have not yet been explored for cellular effects, which was therefore the aim of the present study.

In our laboratory, a series of 3-(2-oxoethylidene)indolin-2-ones were previously synthesized and investigated for their chemopreventive and anti-inflammatory potential [39]. Of note, these compounds are also known as supercinnamaldehyde (SCA) compounds in reference to the parent compound 1 [1-methyl-3(2-oxopropylidene)indolin-2-one] that is commercially known as supercinnamaldehyde. Among the tested compounds, analogs carrying N-substituents were deemed unsuitable candidates for use in chemopreventive and anti-inflammatory strategies due to their strong anti-proliferative properties [39]. Several of the compounds were also found to activate the nuclear factor erythroid 2 p45-related factor 2 (Nrf2) transcription factor [39] and since several Nrf2 activating compounds are known to target TrxR [40], we here hypothesized that Michael acceptor-containing indolin-2-one compounds, particularly those that displayed potent cytotoxicities, could possess TrxR inhibitory activities. In this study, we report the discovery of indolin-2-one compounds carrying *N*-substituents that indeed display enhanced TrxR inhibitory and cellular anti-proliferative activities. *N*-butyl and *N*-benzyl substituents yielded the most potent analogs that could thereby serve as promising lead compounds for development of novel anticancer drug therapies.

## RESULTS

### Indolin-2-one compounds display anti-proliferative and *in vitro* TrxR inhibitory activities

A series of analogs (structures presented in Table 1) containing a Michael acceptor-based 3-(2-oxopropylidene)indolin-2-one scaffold was evaluated for cytotoxicity towards human-derived colorectal HCT 116 and breast MCF-7 carcinoma cells over a duration of 72 h. The compounds exhibited varied growth inhibitory and cytotoxic effects in both cell lines, as indicated by their respective GI_50_ and LC_50_ values (Table 2). HCT 116 cells were generally more susceptible to the compounds. Among the tested compounds, analogs with an R_1_ substituent more bulky than a methyl group (compounds 2-5) were observed to elicit greater anti-proliferative activities. Notably, compounds 5 and 4 bearing a benzyl and *n*-butyl R_1_ substituent, respectively, possessed the strongest anti-proliferative activities (Table 2; compounds were arranged in ascending order of GI_50_ and LC_50_ values). Compound 6, a derivative bearing a methyl and a phenyl substituent at the R_1_ and R_2_ position, respectively, exhibited enhanced growth inhibitory properties in comparison to the parent compound 1. On the other hand, the remaining analogs containing a common methyl R_1_ group and a substituted phenyl group at the R_2_ position did not exhibit enhanced anti-proliferative activities in comparison to the parent compound 1.

**Table 1:** Chemical structures of 3-(2-oxoethylidene)indolin-2-one compounds investigated in this study

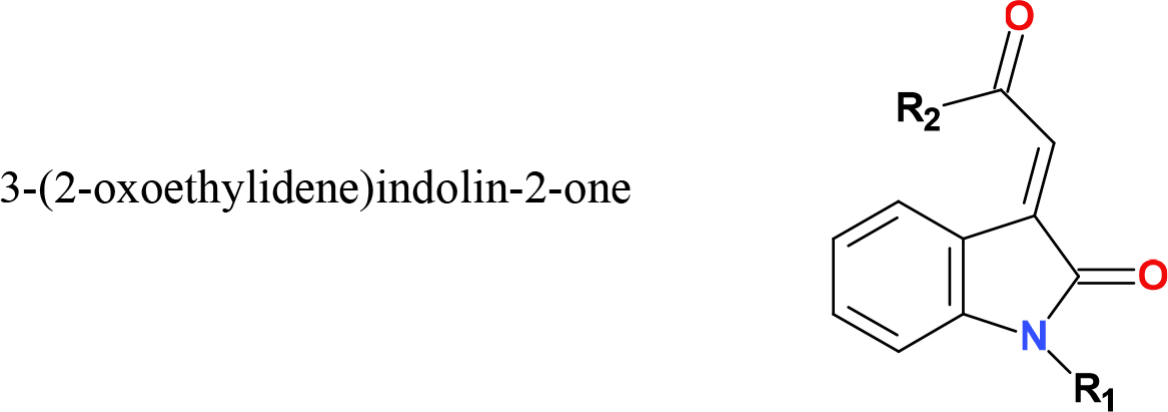					
Compounds	R_1_	R_2_	Compounds	R_1_	R_2_
1^*^	methyl	methyl	**11**	methyl	4-isopropylphenyl
**2**	ethyl	methyl	**12**	methyl	4-*n*-butylphenyl
**3**	isopropyl	methyl	**13**	methyl	4-fluorophenyl
**4**	*n*-butyl	methyl	**14**	methyl	3-chlorophenyl
**5**	benzyl	methyl	**15**	methyl	4-chlorophenyl (E form)
**6**	methyl	phenyl	**16**	methyl	2-(trifluoromethyl)phenyl
**7**	methyl	2-methylphenyl	**17**	methyl	3-(trifluoromethyl)phenyl
**8**	methyl	3-methylphenyl	**18**	methyl	4-(trifluoromethyl)phenyl
**9**	methyl	4-methylphenyl	**20**	methyl	4-chlorophenyl (Z form)
**10**	methyl	4-ethylphenyl			

* Compound 1 is the parent compound; also commercially known as supercinnamaldehyde (SCA).

**Table 2:** Anti-proliferative and TrxR inhibitory activities of indolin-2-one compounds obtained from MTT cell viability assay and *in vitro* TrxR DTNB reduction assay respectively

Compounds	Cell viability				TrxR inhibition
	HCT 116 cells		MCF-7 cells		30 min
	GI_50_ (μM)	LC_50_ (μM)	GI_50_ (μM)	LC_50_ (μM)	IC_50_ (μM)
**5**	3.2 ± 0.1	9.4 ± 0.3	4.5 ± 0.6	9.6 ± 0.2	6.2 ± 1.7
**4**	4.5 ± 0.9	9.7 ± 0.2	6.4 ± 1.5	22.4 ± 3.6	8.6 ± 0.9
**2**	4.2 ± 0.5	9.7 ± 0.1	7.9 ± 1.7	25.0 ± 1.6	10.2 ± 1.8
**3**	5.3 ± 0.1	9.8 ± 0.1	7.1 ± 0.6	22.4 ± 0.6	15.0 ± 1.4
**6**	8.5 ± 0.8	27.2 ± 2.4	14.0 ± 3.7	27.1 ± 0.5	14.7 ± 3.2
**8**	10.4 ± 2.6	28.0 ± 1.0	14.0 ± 0.6	27.9 ± 0.6	22.5 ± 0.4
**9**	10.5 ± 1.2	28.6 ± 0.8	10.5 ± 0.8	28.4 ± 0.8	21.6 ± 4.4
**1**	11.8 ± 3.3	26.8 ± 1.2	15.1 ± 4.1	27.6 ± 0.4	22.1 ± 1.8
**10**	11.9 ± 1.1	28.8 ± 1.1	10.4 ± 0.6	30.4 ± 6.2	19.1 ± 0.4
**13**	11.9 ± 0.5	29.0 ± 1.4	12.5 ± 2.4	28.6 ± 1.2	18.7 ± 3.56
**15**	12.4 ± 2.8	28.6 ± 1.4	20.9 ± 3.0	92.0 ± 1.2	16.9 ± 3.4
**7**	12.4 ± 2.1	27.8 ± 0.8	17.3 ± 4.6	27.9 ± 1.5	19.0 ± 2.4
**12**	13.5 ± 2.6	26.7 ± 0.8	16.5 ± 4.0	81.2 ± 9.7	> 100
**11**	14.4 ± 1.8	26.7 ± 1.7	10.5 ± 1.7	27.0 ± 0.4	25.2 ± 5.4
**14**	15.6 ± 1.1	26.9 ± 1.5	23.0 ± 4.5	84.3 ± 5.8	18.4 ± 2.2
**18**	15.7 ± 1.7	90.5 ± 7.3	44.4 ± 0.1	89.8 ± 5.1	> 100
**17**	16.1 ± 2.2	26.2 ± 2.1	17.9 ± 0.4	89.6 ± 3.6	34.2 ± 18.2
**20**	16.1 ± 2.4	28.7 ± 2.4	20.4 ± 3.5	91.7 ± 2.0	15.9 ± 2.9
**16**	19.5 ± 1.3	27.7 ± 3.1	15.8 ± 1.0	27.7 ± 1.9	28.9 ± 0.2

Compounds were listed in descending order of their anti-proliferative activities against HCT 116 cells.

Values for 50% growth inhibition concentration (GI_50_), 50% lethal concentration (LC_50_), and 50% inhibition concentration (IC_50_), are presented as means ± SD of 3 independent experiments.

**Figure 1:**
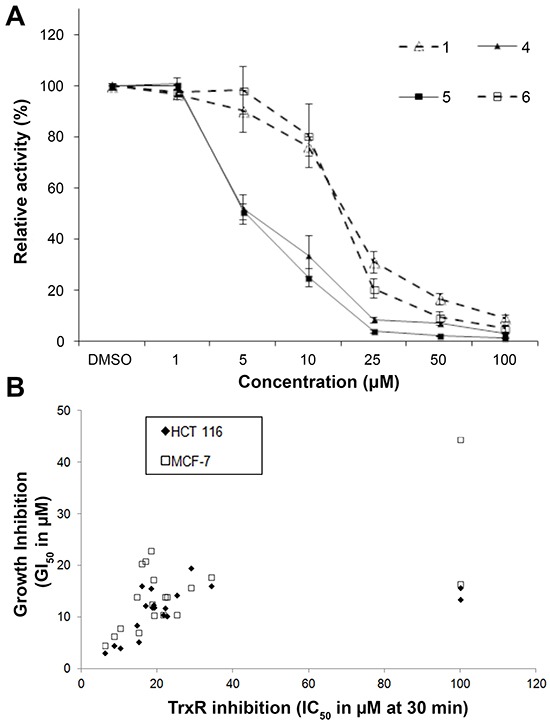
*In vitro* effects of compounds 1, 4, 5 and 6 on recombinant rat TrxR activity and correlation of the *in vitro* TrxR inhibitory and anti-proliferative potencies of indolin-2-one compounds **A.** TrxR activity was evaluated by DTNB reduction assay after 30 min incubation of the indicated compounds with 100 nM recombinant rat TrxR and 200 μM NADPH. All data points are means ± SD of at least 2 independent experiments. **B.** The *in vitro* 50% TrxR inhibition concentration (IC_50_) values at 30 min were plotted against the corresponding growth inhibition concentration (GI_50_) values of the tested indolin-2-one compounds obtained in HCT 116 and MCF-7 cells. A strong and fair correlation between the TrxR inhibitory and anti-proliferative activities of the tested analogs (excluding outliers compounds 12 and 18) was obtained for the HCT 116 and MCF-7 cell line respectively. Calculated linear correlation coefficient r values are 0.80 (for HCT 116 cells) and 0.47 (for MCF-7 cells).

The inhibition of recombinant mammalian TrxR by indolin-2-one compounds after 30 min of incubation in the presence of NADPH was evaluated in the *in vitro* 5,5′-dithiobis(2-nitrobenzoic acid) acid (DTNB) reduction assay and the IC_50_ values are presented in Table 2. To illustrate the dose-dependent inhibitory activities of the tested compounds, the extent of TrxR inhibition by selected indolin-2-one compounds 1, 4, 5 and 6 over a range of 1-100 μM is presented in Figure 1A. Comparison of the IC_50_ values at 30 min with the GI_50_ values revealed a strong correlation between the TrxR inhibitory and anti-proliferative activities of the analogs for the HCT 116 cell line (r = 0.8) whereas for the MCF-7 cell line, the correlation was relatively weaker (r = 0.47) (Figure 1B). This correlation suggested that TrxR inhibition could potentially serve as an underlying mechanism for at least part of the anti-proliferative effects of these compounds. The effects of lead indolin-2-one compounds 4 and 5 on the viability of MRC-5 human normal lung fibroblasts were also tested and found to possess a greater LC_50_ value by approximately 2-fold (compound 4: LC_50_ value 9.7 ± 0.2 μM in HCT 116 cells versus 25.5 ± 2.7 μM in MRC-5 cells; compound 5: LC_50_ value 9.4 ± 0.3 μM in HCT 116 cells versus 22.7 ± 2.4 μM in MRC-5 cells. The marginal selectivity of the compounds for cancer cell lines over normal cell types would need further work either through deriving more analogs bearing desirable structural features or utilization of cancer cell-targeted delivery approaches to improve selectivity.

### Lead indolin-2-one compounds selectively inhibit cellular TrxR activity

**Figure 2:**
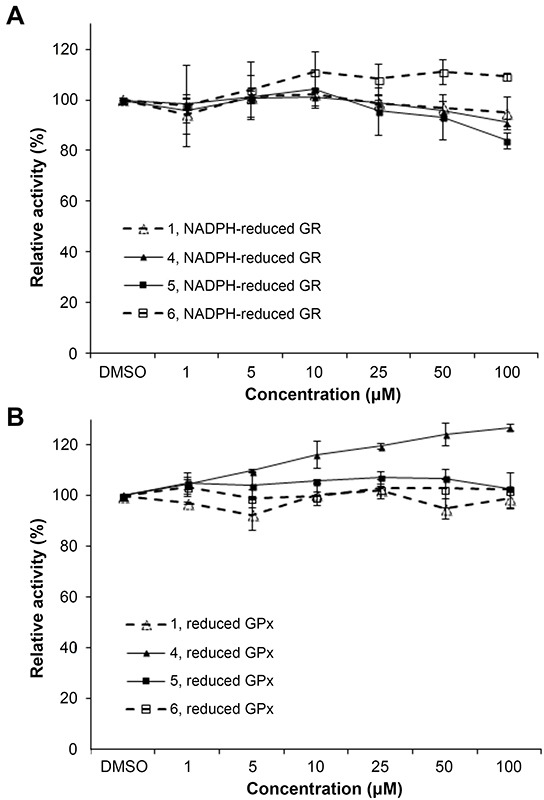
*In vitro* effects of compounds 1, 4, 5 and 6 on yeast GR and bovine GPx activity **A.** The activity of yeast GR (20 nM) incubated with the indicated compounds and 200 μM NADPH for 1 h was determined by following the absorbance at 340 nm upon addition of GSSG. **B.** The activity of bovine GPx (100 nM) incubated with the indicated compounds, 20 nM yeast GR, 200 μM NADPH and 1 mM GSH for 1 h was determined by measuring the absorbance at 340 nm upon addition of H_2_O_2_. All data points were means ± SD of 2 independent experiments.

**Figure 3:**
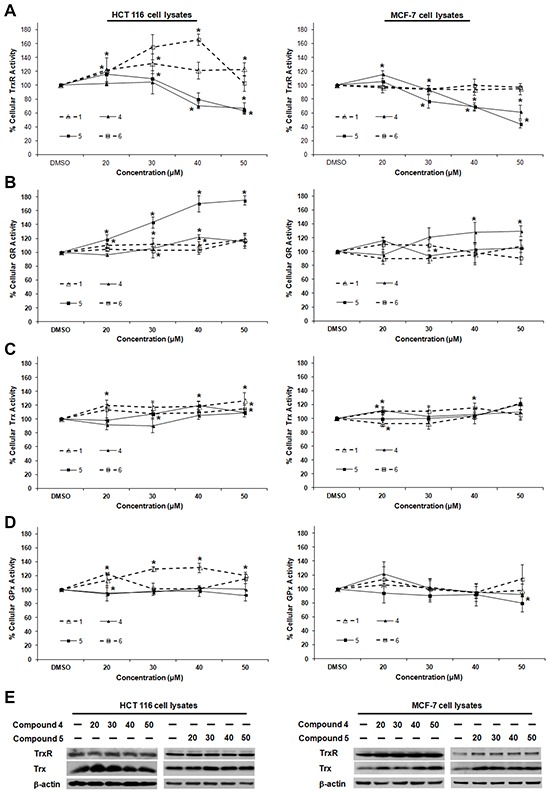
Dose-dependent effects of compounds 1, 4, 5 and 6 on TrxR, GR, Trx and GPx activities in HCT 116 and MCF-7 cells Lysates of HCT 116 and MCF-7 cells treated with indicated concentrations of selected SCA analogs for 10 h were assessed for activities of **A.** TrxR, **B.** GR, **C.** Trx and **D.** GPx. Enzyme activities were expressed as a percentage of those in DMSO-treated cells. All data points are means ± SD of two to four independent experiments. * Statistically significant difference (*p* < 0.05) in enzyme activity as compared to DMSO control. **E.** The lysates of cells treated with compound 4 and 5 as mentioned in (A)-(D) were analyzed by Western blotting with indicated antibodies for TrxR and Trx expression. Western blot images are representative of three independent experiments.

**Figure 4:**
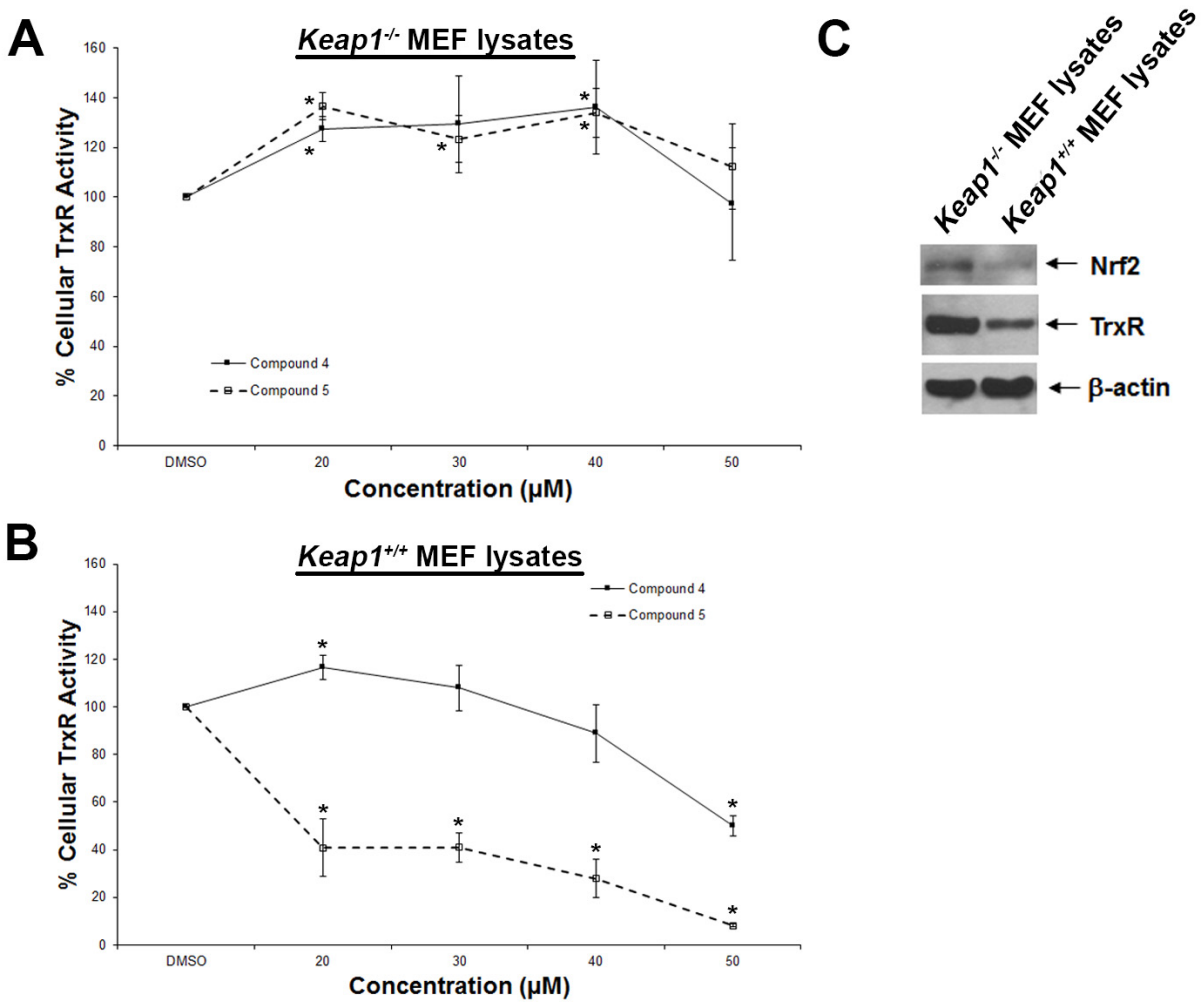
Dose-dependent effects of compounds 4 and 5 on TrxR activity in wild-type and Keap1-null MEFs Lysates of **A.** Keap1-null (*Keap1^−/−^*) and **B.** wild-type (*Keap1^+/+^*) MEFs treated with indicated concentrations of compounds 4 and 5 for 5 h were assessed for TrxR activities. **C.** Lysates of untreated *Keap1^−/−^* and *Keap1^+/+^* MEFs were analyzed by Western blotting with indicated antibodies for Nrf2 and TrxR expression. Western blot images are representative of three independent experiments.

To further probe whether the correlation between TrxR inhibition and cytotoxic effects (Figure 1B) could be casual, we next assessed whether the compounds were more selective for TrxR than targeting other redox active enzymes. Specificity of the selected indolin-2-one compounds 1, 4, 5 and 6 towards GR and GPx was first evaluated in *in vitro* assays using pre-reduced yeast non-selenoprotein GR and bovine selenoprotein GPx enzymes. As shown in Figure 2A and 2B respectively, activities of yeast GR and bovine GPx were found to be uninhibited after 60 min incubation with the selected compounds. Instead, some compounds were observed to cause marginal elevation of GR (for compound 6) and GPx (for compound 4) activity. The selectivity of these four analogs against Trx- and GSH-related enzymes was next examined in a cellular context using HCT 116 and MCF-7 cells. As illustrated in Figure 3A, a shorter 10 h treatment with compounds 1 and 6 at concentrations around the LC_50_ values in the 72 h incubations (20, 30 and 40 μM; see Table 1) and a lethal concentration (50 μM) did not cause apparent inhibition of cellular TrxR activity. On the contrary, the more cytotoxic lead compounds 4 and 5 caused a decrease in TrxR activity in a dose-dependent manner within this time frame (Figure 3A). In particular, compound 5 produced a significant reduction in cellular TrxR activity at 40 and 50 μM, respectively, in both HCT 116 and MCF-7 cells (Figure 3A). Additionally, Western blot analyses showed that levels of TrxR protein in lysate samples of these cells treated with compounds 4 and 5 were not lower than in cells treated with vehicle, suggesting that the decrease in cellular TrxR activity was due to formation of irreversibly inhibited enzyme species (Figure 3E). The cellular activities of GR, Trx and GPx, in contrast, were either constant or increased (Figure 3B, 3C and 3D respectively), indicating that the indolin-2-one compounds were selective for TrxR inhibition. The increased activities could likely have been due to Nrf2 activation, as previously found for several indolin-2-one analogs [39]. Indeed, when wild-type (*Keap1^+/+^*) and Keap1-null (*Keap1^−/−^*) MEFs were treated with compounds 4 and 5, the *Keap1^−/−^* MEFs that had the *Keap1* gene {Keap1 protein is a negative regulator of Nrf2 [41]} knocked out were not only more resistant to indolin-2-one-mediated TrxR inhibition (Figure 4A) than the *Keap1^+/+^* MEFs (Figure 4B), but also displayed elevated TrxR activity when treated with compounds at concentrations 20 to 40 μM (Figure 4A). The results, which were in agreement with higher expression levels of Nrf2 and TrxR in the *Keap1^−/−^* MEFs than those in the *Keap1^+/+^* MEFs (Figure 4C), had indicated that the increased activities of TrxR in indolin-2-one-treated cells were attributed to increased Nrf2 activation.

### TrxR inhibition by lead indolin-2-one compounds leads to Trx oxidation and induction of ASK1-mediated apoptotic cell death

**Figure 5:**
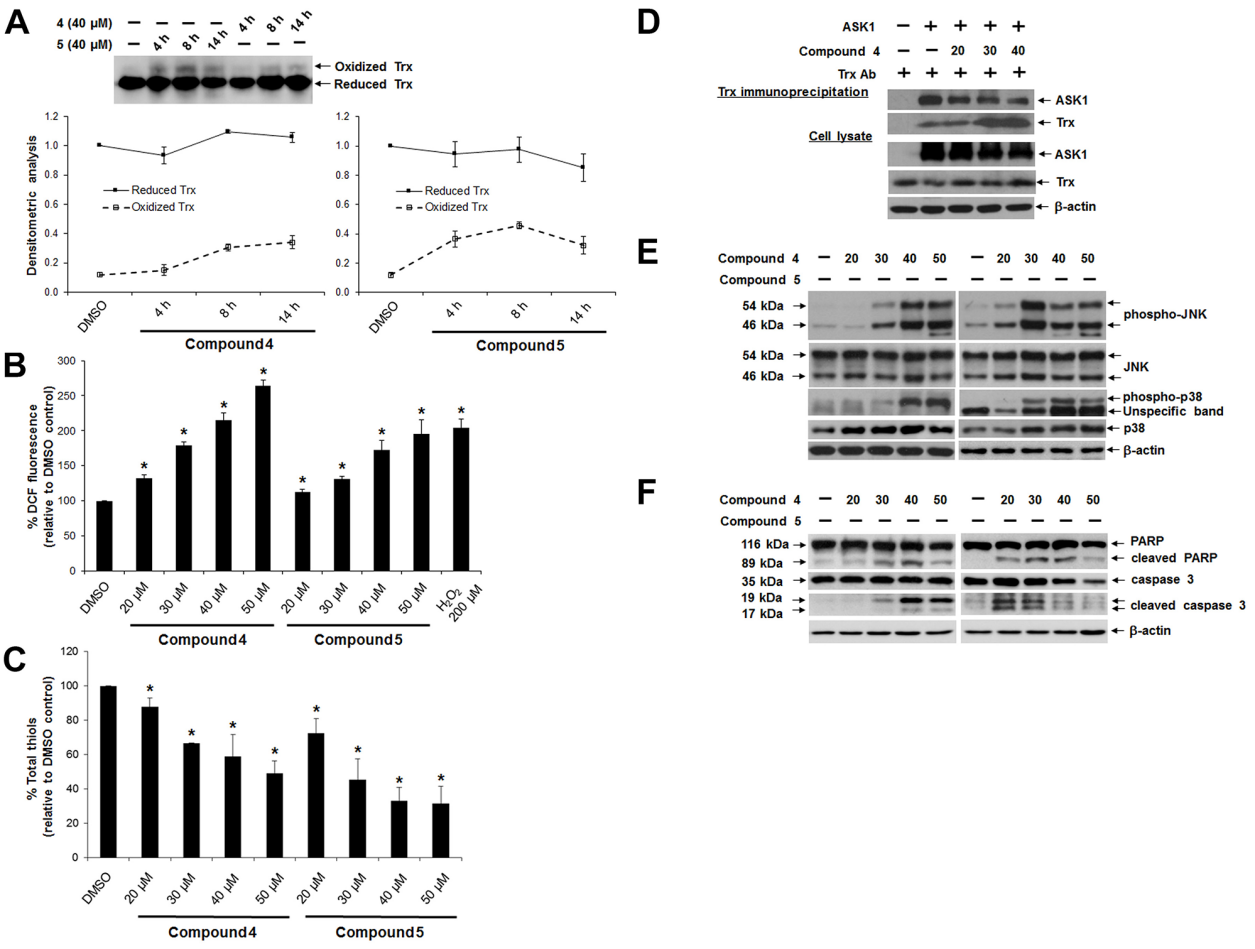
Effects of lead indolin-2-one compounds on Trx oxidation, Trx-ASK1 interaction and apoptosis induction in HCT 116 cells **A.** *Top panel:* time-dependent Trx oxidation caused by treatment with indolin-2-one compounds. Lysates of cells treated with compound 4 or 5 (40 μM) for 4, 8 and 14 h were collected in guanidine-containing lysis buffer containing 50 mM IAA and subjected to native PAGE and Western blot analysis using Trx antibody. *Bottom panel:* mean densitometric intensities of reduced and oxidized Trx protein bands for each treatment group (n = 3). **B.** Dose-dependent increase in DCF fluorescence in cells treated with indolin-2-one compounds. Following a 3-h treatment with compound 4 or 5 at indicated concentrations, HCT 116 cells were incubated with 10 μM carboxy-H_2_DCFDA in the dark for 45 min at 37°C. Fluorescence was then measured at 530 nm. * Statistically significant difference (*p* < 0.05) as compared to DMSO control (n = 3). **C.** Effects of indolin-2-one compounds on total cellular thiol levels. Following a 4-h treatment with compound 4 or 5, total thiol content in HCT 116 cells was assessed. * Statistically significant difference (p < 0.05) as compared to DMSO control (n = 3). **D.** Effects of indolin-2-one compound on Trx-ASK1 interaction. Lysates from ASK1-transfected HCT 116 cells after 2-h treatment with DMSO or compound 4 were subjected to immunoprecipitation using anti-human Trx antibody. Immunoprecipitates and aliquots of the cell lysates were subjected to SDS-PAGE and Western blot analysis using the indicated antibodies. **E.** Dose-dependent effects of indolin-2-one compounds on MAPK activation. Lysates of cells after 3 h of treatment with DMSO, compound 4 or 5 were collected and subjected to SDS-PAGE and Western Blot analysis of levels of phosphorylated and total JNK and p38. **F.** Dose-dependent induction of apoptosis, indicated by presence of cleaved caspase 3 (17/19 kDa) and PARP (89 kDa) in HCT 116 cells treated with compound 4 or 5 for 10 h. All Western blot images shown are representative of three independent experiments.

The cellular events linking TrxR inhibition by indolin-2-one lead compounds to cell death were examined in HCT 116 cells. First, the redox state of cellular Trx was probed using an IAA-coupled alkylation method [42]. This analysis had revealed time-dependent oxidation of Trx following treatment with compounds 4 and 5 at the lethal dose of 40 μM (Figure 5A), suggesting that TrxR inhibition caused by indolin-2-one compounds triggered Trx oxidation. Second, a significant dose-dependent elevation in cellular oxidative stress was evident within 3 h of treatment with compounds 4 and 5, as illustrated using a DCF fluorescence assay (Figure 5B). Notably, treatment with 50 μM of compound 4 and 5 resulted in markedly increased DCF fluorescence as compared to that seen in DMSO control cells. Consistent with the observed increase in DCF fluorescence, treatment of HCT 116 cells with lead compounds 4 and 5 for 5 h were found to cause depletion of total cellular thiols in a dose-dependent manner (Figure 5C). This had indicated the reactivity of indolin-2-one compounds to thiols and the more oxidized state of the cells as compared to untreated cells.

One mechanism through which Trx can exert anti-apoptotic effects involves suppression of ASK1-dependent apoptosis through a direct inhibitory protein-protein interaction between reduced Trx and ASK1 [43]. This interaction is dependent on the redox state of Trx, with oxidation of Trx resulting in the dissociation of the Trx-ASK1 complex, which was therefore examined next. Indeed, Trx immunoprecipitation from lysates of ASK1-overexpressing HCT 116 cells treated with compound 4 revealed a dose-dependent decrease in the amount of ASK1 bound to Trx1 (Figure 5D). This corresponded to an increased activation of the ASK1 downstream mitogen-activated protein kinases (MAPKs) targets p38 and JNK [phosphorylated p38 (p-p38) and JNK (p-JNK)] (Figure 5E). Finally, ASK1-dependent activation of p38 and JNK MAPK pathways triggered apoptotic cell death, as evident from a dose-dependent appearance of apoptotic markers such as cleaved forms of PARP and caspase 3 (Figure 5F). Of note, a higher concentration (50 μM) of the more cytotoxic compound 5 brought about lower activation of PARP and caspase-3 (Figure 5F), which could have been due to excessive oxidative stress instead leading to necrosis.

### Lead indolin-2-one compounds inhibit TrxR irreversibly and target the penultimate selenocysteine residue in the C-terminal active site

**Figure 6:**
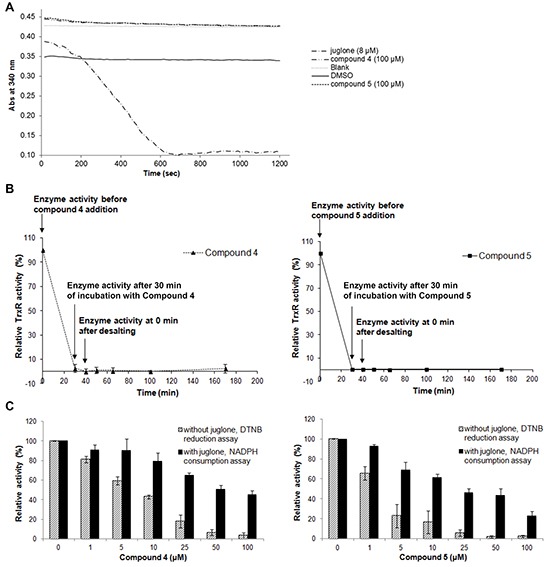
Effects of lead indolin-2-one compounds on NADPH oxidase activity of recombinant rat TrxR, irreversibility of TrxR inhibition, and juglone and DTNB reduction by TrxR **A.** Upon incubation of recombinant rat TrxR (15 nM) with 250 μM NADPH and juglone, compound 4 or 5 at indicated concentrations, consumption of NADPH was measured at 340 nm for 20 min. Results represent the mean ± SD of three independent experiments. **B.** Investigation of the irreversibility of TrxR inhibition by compounds 4 and 5. Recombinant rTrxR (0.9 μM) was incubated with 20 μM of the lead compounds and 200 μM NADPH for 30 min. Excess compound was removed by desalting the protein sample using an NAP-5 column. TrxR activity was determined by DTNB reduction assay at indicated timepoints before and after desalting. Results shown are representative of two independent experiments. **C.** Effects of lead indolin-2-one compounds on dose-dependent juglone reduction and DTNB reduction by TrxR. Recombinant rat TrxR (15 nM) was reduced by 250 μM NADPH and incubated with indicated concentrations of compound 4 or 5 for 15 min. Following the incubation, DTNB reduction or juglone reduction were measured. For DTNB reduction, 50 μl of 8.75 μM DTNB solution in TE buffer was added and DTNB reduction was measured at 412 nm for 2 min. For juglone reduction, 100 μl of 300 μM juglone solution in TE buffer was added and NADPH consumption at 340 nm was monitored for 5 min. The results were expressed as % of TrxR juglone reduction or DTNB reduction activity of drug-treated sample over that of DMSO-treated sample. Results are presented as means ± SD of three independent experiments.

The mode of TrxR inhibition by lead indolin-2-one compounds was examined in more detail. Firstly, compounds 4 and 5 were evaluated as substrates of TrxR. As shown in Figure 6A, negligible increase in the rate of NADPH oxidation was observed upon addition of the compounds, as compared to that of DMSO-treated enzyme. On the contrary, the rate of NADPH consumption of recombinant TrxR treated with juglone, a known TrxR substrate [44], was markedly increased (Figure 6A). This indicated that the compounds were not TrxR substrates. Secondly, the reversibility of the TrxR inhibition by compounds 4 and 5 was investigated by treating NADPH-reduced recombinant TrxR with 20 μM of the compounds for 30 min to fully abolish its activity, followed by desalting to remove any excess of unbound compound. TrxR activity could not be recovered within 2 h of post-desalting (Figure 6B), suggesting that compounds 4 and 5 were irreversible inhibitors of TrxR.

**Figure 7:**
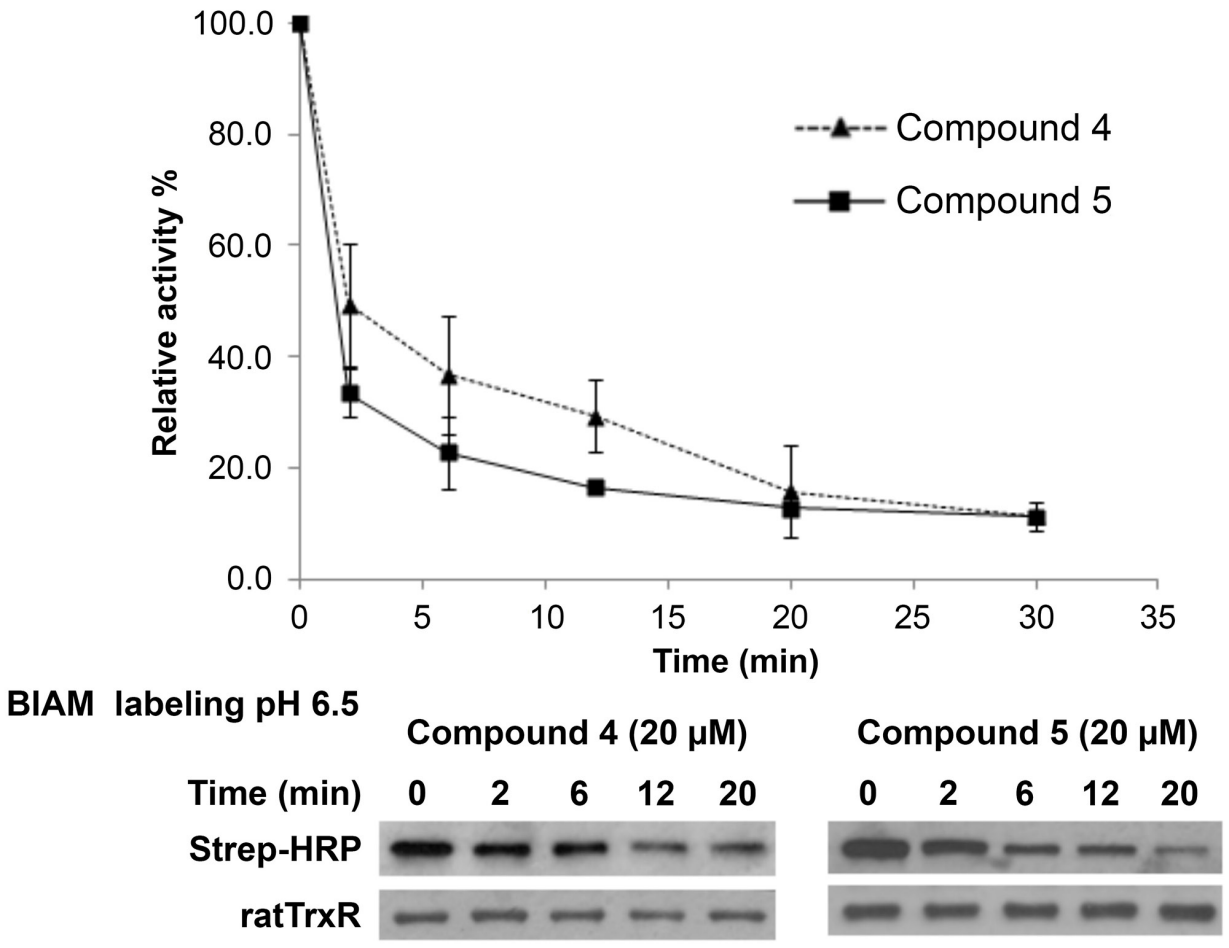
Investigation of the interaction of lead indolin-2-one compounds with the Sec residue in the C-terminal active center of TrxR Recombinant rat TrxR (0.9 μM) was incubated with 20 μM of the lead compounds and 200 μM NADPH. At indicated timepoints, an aliquot of enzyme mixture was drawn for TrxR activity measurement by DTNB reduction assay and BIAM labelling at pH 6.5. *Top panel:* time course of TrxR enzyme activity; *bottom panel:* horseradish peroxidase (HRP)-conjugated streptavidin detection of BIAM labeling of free selenol at pH 6.5 at various incubation times. Results presented are representative of three independent experiments.

Several inhibitors of mammalian TrxR have been shown to cause formation of selenium compromised thioredoxin reductase apoptotic proteins (SecTRAPs), which are incapable of supporting Trx reduction due to targeting of the Sec residue in the C-terminal active center, whereas an N-terminal active center remains functional allowing for an NADPH oxidase-like activity of the enzyme to persist [45]. Generation of SecTRAPs can thereby lead to enhanced ROS production, which may trigger a mixture of apoptotic and necrotic cell death due to excessive oxidative stress [45]. To investigate whether the compounds produced SecTRAPs from TrxR, redox cycling with juglone by recombinant TrxR inhibited by lead indolin-2-one compounds was studied, which is a model assay for SecTRAPs properties [45]. The results showed that juglone reduction was more preserved than DTNB reduction upon inhibition by compounds 4 and 5 (Figure 6C), indeed suggesting formation of SecTRAPs. We next investigated whether the compounds targeted the Sec residue in the C-terminal active center of mammalian TrxR using an alternative N-(biotinoyl)-N′-(iodoacetyl)-ethylenediamine (BIAM) labeling assay [46]. In this assay, recombinant TrxR incubated with 200 μM NADPH and 20 μM of compound 4 or 5 for specified timepoints was next probed with BIAM at pH 6.5, which allows preferential alkylation of reactive Sec residues but not Cys residues due to the difference in pKa between Sec (pKa 5.2) and Cys (pKa 8.0). Treatment with compounds 4 or 5 resulted in a decrease in TrxR activity over time (top panel of Figure 7), which correlated with a prevention of BIAM alkylation (bottom panel of Figure 7).

## DISCUSSION

We previously reported that hydroxy and fluorine substitutions render strong TrxR inhibitory character to cinnamaldehydes and compounds containing a 1,5-diphenyl-pent-1-en-3-one (DDPen) or 1,3-diphenyl-prop-1-en-3-one (DPPro; also known as chalcone) pharmacophore [47, 48]. In the present study, the evaluated indolin-2-one compounds contain the 3-(2-oxopropylidene)indolin-2-one backbone, which possesses two conjugated α,β-unsaturated carbonyl groups as Michael acceptors. We found a majority of the compounds displayed a good correlation between anti-proliferative activities toward two human cancer cell lines and TrxR inhibition, which has several important implications.

It is notable that several of the indolin-2-one compounds studied here are also Nrf2 inducers [39], which agrees well with the notion that TrxR inhibition can lead to Nrf2 activation, as recently discussed elsewhere in further detail [40]. Our assessments of the compounds' effects on TrxR, Trx, GR and GPx had both demonstrated target selectivity, as only TrxR was among these enzymes inhibited by the compounds *in vitro*, and Nrf2 activation, as the cellular activities of these other Nrf2-induced enzymes were increased in a cellular context together with inhibition of TrxR.

As the indolin-2-one compounds irreversibly inhibited TrxR, this suggested that covalent binding occurred between the compounds and a nucleophilic Sec, or possibly Cys residue in TrxR. The sustained juglone reduction activity of the inhibited enzyme, as well as the BIAM assay results, were findings that would be compatible with preferential targeting at the Sec residue of TrxR. This effect can also explain the cytotoxicity of these compounds, most likely being due to a rapidly triggered oxidative stress resulting in both specific signaling events leading to apoptosis as well as necrotic cell death.

We propose that oxidative stress upon indolin-2-one-mediated TrxR inhibition can be triggered *via* several specific pathways. Firstly, as inferred from results obtained from the juglone reduction assay, treatment with compounds 4 and 5 can result in formation of SecTRAPs, thereby converting TrxR to a prooxidant NADPH oxidase. Indeed, as it had been observed previously for 1-chloro-2,4,-dinitrobenzene (DNCB) or cisplatin, the formation of SecTRAPs in cells can result in rapid induction of oxidative stress leading to cell death [45, 49]. Secondly, increased levels of oxidized Trx (as a result of TrxR inhibition) should prevent Trx of providing reducing equivalents to peroxiredoxins, which will further aggravate the oxidative stress as a result of impaired antioxidant defense. Furthermore, our results were compatible with the effects of increased Trx oxidation leading to dissociation of Trx-ASK1 complexes, followed by activation of ASK1 and its downstream JNK and p38 MAPK signaling pathways leading to apoptotic cell death induction [43, 50]. Indeed, dissociation of the ASK1-Trx complex in HCT 116 cells upon treatment with compound 4 was observed in our study. As treatment with lead indolin-2-one compounds also resulted in a dose-dependent increase in levels of activated p38 and JNK, this showed involvement of these two MAPK signaling cascades in mediating cell death induced by these compounds.

**Figure 8:**
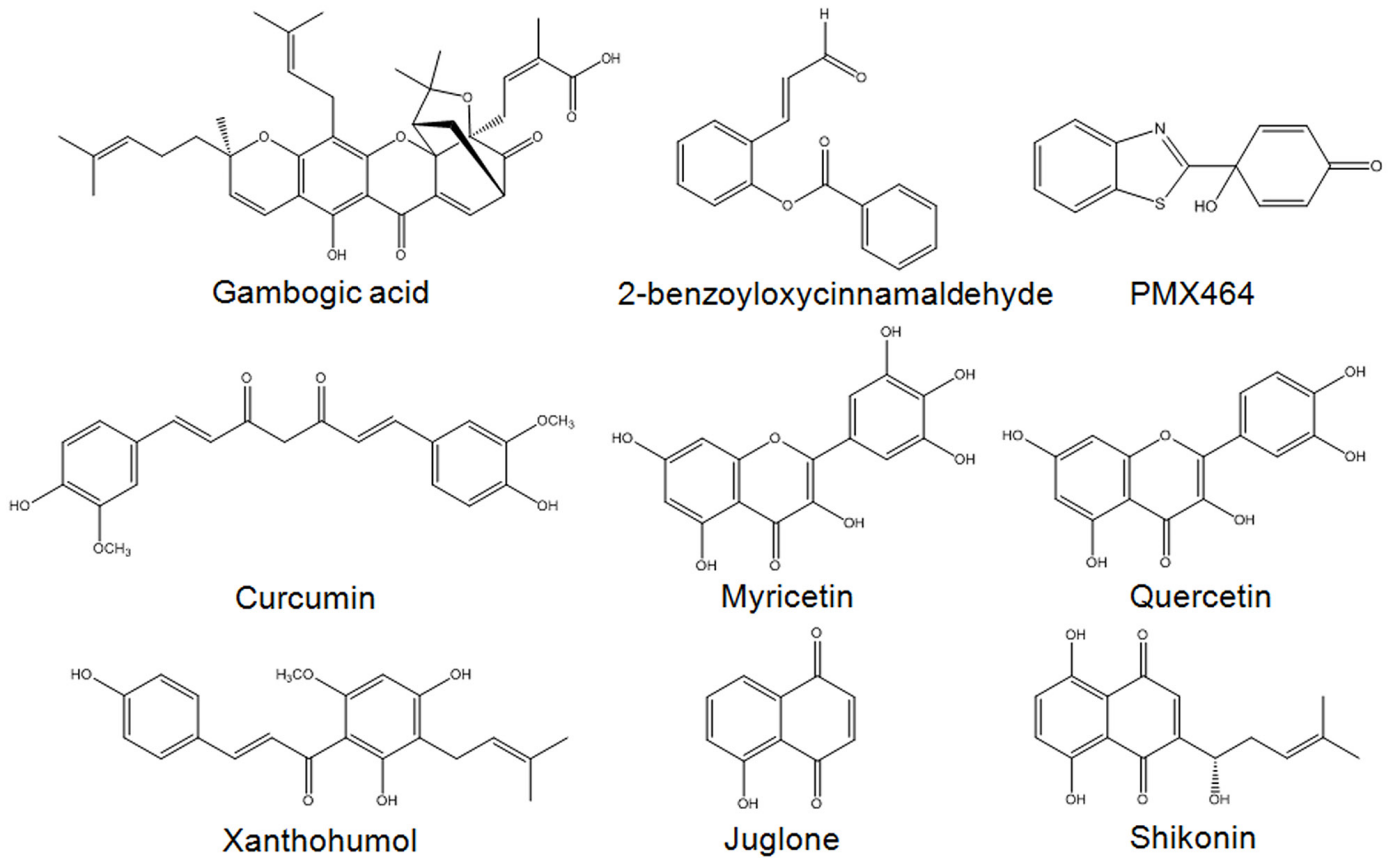
Examples of Michael acceptor moiety-containing compounds reported to possess TrxR inhibitory activities Compounds include nature-inspired gambogic acid [51], ortho-substituted cinnamaldehydes such as 2-benzoyloxycinnamaldehyde (BCA) [47], curcumin [52], flavonoids myricetin and quercetin [53], chalcone-containing xanthohumol [54], quinone compounds juglone [44] and shikonin [55], as well as quinol compound PMX464 [56].

**Figure 9:**
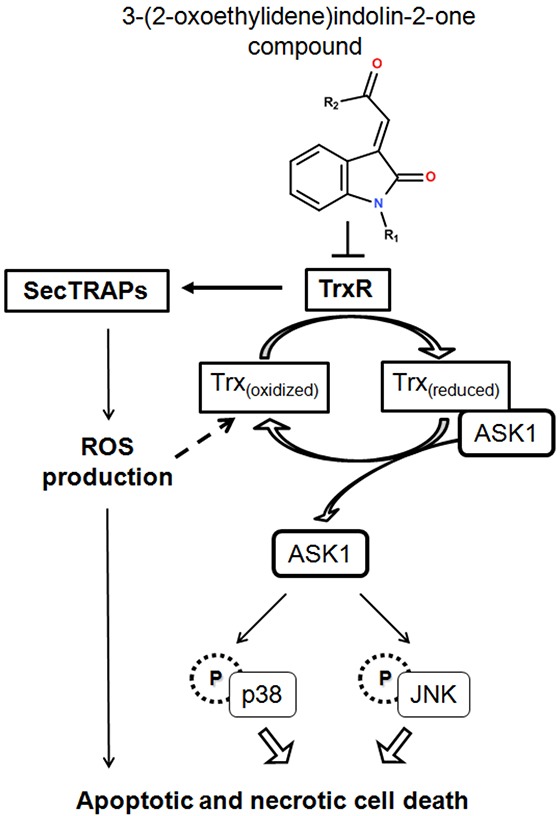
A summary of cellular events resulting from TrxR inhibition caused by treatment with indolin-2-one compounds TrxR inhibition by indolin-2-one compounds led to formation of SecTRAPs and Trx oxidation. SecTRAPs lacking Trx reducing activity of TrxR could lead to enhanced ROS production and trigger a mixture of apoptotic and necrotic cell death. Trx oxidation further led to dissociation of ASK1 from Trx_(reduced)_-ASK1 complexes, followed by ASK1-dependent activation of MAPK signaling pathways, resulting in cell death.

In summary, Michael acceptor-based compounds are a class of TrxR inhibitors that possess electrophilicities to target nucleophilic Sec and Cys residues in the active sites of mammalian TrxR. Examples of several reported TrxR inhibitors that bear a Michael acceptor moiety [44, 47, 51–56] are illustrated in Figure 8. This study has evaluated a series of Michael acceptor-based indolin-2-one compounds and identified lead compounds 4 and 5 carrying a *N*-butyl and *N*-benzyl substituent, respectively, to display the strongest TrxR inhibitory and anti-proliferative activities. Their selectivity towards inhibition of cellular TrxR activity was mediated through targeting the Sec residue in the enzyme's C-terminal active site. Mechanistically, the cytotoxicities of these lead indolin-2-one analogs were mediated at least in part through TrxR inhibition and Trx oxidation, followed by ASK1-dependent activation of p38 and JNK MAPK signaling pathways (summarized in Figure 9). Importantly, this study has provided experimental evidence attesting that appropriate structural modifications to compounds bearing the indolinone chemical scaffold, particularly substitutions at the nitrogen atom, can yield derivatives that possess specific TrxR inhibitory activity as leads for anticancer drug development.

## MATERIALS AND METHODS

### Materials, chemicals and cell culture

Recombinant rat TrxR was prepared as previously described [57]. Recombinant human Trx (rhTrx1) and anti-human Trx antibody were obtained from IMCO Corporation (Stockholm, Sweden). Iodoacetamide (IAA), yeast glutathione reductase (GR), bovine glutathione peroxidase (GPx), glutathione disulfide (GSSG), reduced glutathione (GSH) and trypsin of proteomics grade were purchased from Sigma. 5-(and-6)-carboxy-2′,7′-dichlorodihydrofluorescein diacetate (carboxy-H_2_DCFDA), BIAM and HRP-conjugated streptavidin were from Molecular Probes and bovine insulin was from Gemini Bio-Products. Antibodies against Nrf2, full-length and cleaved caspase 3, full-length and cleaved poly (ADP)-ribose polymerase (PARP), JNK, phospho-JNK (Thr183/Tyr185), p38 and phospho-p38 (Thr180/Tyr182) were from Cell Signaling Technology, while those specific for human TrxR1, ASK1 and β-actin and HRP-conjugated secondary antibodies were from Santa Cruz Biotechnology. Human-derived colon HCT 116 and MCF-7 breast carcinoma cells were maintained in RPMI 1640 medium, MRC-5 normal lung fibroblasts in MEM and wild-type (*Keap1^+/+^*) and *Keap1^−/−^* MEFs were cultured in IMDM containing epidermal growth factor (EGF; 1 μg per 100 ml medium). All media were supplemented with 10% fetal bovine serum, 100 units/ml penicillin and 100 μg/ml streptomycin. The cells were incubated at 37°C in an humidified atmosphere of 95% air and 5% CO_2_. Indolin-2-one compounds 1 to 20 were previously synthesized and were all at least 95% pure as assessed by HPLC-MS and 1H NMR [39]. They were kept as 50 mM DMSO stocks at −20°C and diluted fresh in DMSO or culture medium to the final concentrations immediately before all experiments.

### Cell viability assay

HCT 116 and MCF-7 cells were seeded in 96-well plates at a density of 1 × 10^4^ cells/well and allowed to grow for 20-24 h before addition of test compounds. DMSO stocks of indolin-2-one compounds were serially diluted in medium each time an assay was performed. Cell viability was determined at the time of compound addition (time 0) and after 72 h of compound treatment by reduction of 3-(4,5-dimethylthiazol-2-yl)-2,5-diphenyltetrazolium bromide (MTT) by dehydrogenases in viable cells. MTT was added into each well (final concentration 400 μg/ml) and the plates were incubated for 4 h at 37°C. The supernatants were aspirated and upon solubilization of the formed formazan crystals in DMSO:glycine buffer pH 10.5 (ratio 4:1), absorbance at 550 nm was measured. 50% growth inhibition concentrations (GI_50_) and 50% lethal concentrations (LC_50_) values were calculated.

### Determination of recombinant rat TrxR activity by DTNB reduction assay

In a volume of 100 μl of 50 mM Tris-HCl pH 7.5, 2 mM EDTA buffer (TE buffer), compounds of concentrations 1-100 μM were incubated with 100 nM recombinant rat TrxR and 200 μM NADPH at room temperature in 96-well plates for 30 and 60 min. The incubation was terminated by addition of 100 μl per well of 10 mM DTNB, 200 μM NADPH solution in TE buffer and immediately followed by measurement of linear increase of absorbance at 412 nm for the initial 2 min using the VersaMax microplate reader (Molecular Devices). The results were expressed as % of TrxR activity of drug-treated sample over that of DMSO control. The 50% inhibition concentration (IC_50_) values were calculated.

### Determination of juglone reduction by recombinant rat TrxR

The assay, designed to determine the ability of mammalian TrxR to reduce juglone at the N-terminal active center, was performed as described previously [44, 45]. Briefly, 2 μl of diluted DMSO stocks of compounds 4 and 5 (final concentrations 1-100 μM) were incubated in 198 μl of 15 nM recombinant rat TrxR, 250 μM NADPH and 0.1 mg/ml BSA in TE buffer at room temperature in 96-well plates for 15 min. 100 mM stock solution of juglone in DMSO was prepared fresh for each experiment and diluted in TE buffer to a 300 μM working solution. The incubation was terminated by the addition of 100 μl of 300 μM juglone solution (final concentration 100 μM), and NADPH consumption was monitored at 340 nm for 5 min using the VersaMax microplate reader. The linear decrease in NADPH absorbance was used for calculating juglone reduction activity. The results were expressed as % of TrxR juglone reduction activity of drug-treated sample over that of DMSO-treated sample.

The experiments were performed at the same time with a corresponding DTNB reduction assay as described above with modifications so as to keep the experimental parameters and conditions similar to those in the juglone reduction assay. Performing the DTNB reduction assay together with the juglone reduction assay ensured that any amount of TrxR inhibition registered in each assay could be directly cross-compared with each other at the same time. Following 15 min incubation with compound 4 or 5, 50 μl of 8.75 μM DTNB solution (final concentration 1.75 μM) was added to each well and DTNB reduction was measured at 412 nm for 2 min.

### Determination of NADPH oxidase activity of recombinant rat TrxR

In a volume of 198 μl of 15 nM recombinant rat TrxR and 250 μM NADPH in TE buffer, 2 μl of diluted DMSO stocks of juglone and compounds 4 and 5 was added to final concentrations of 8 μM (for julgone) and 100 μM (for compounds 4 and 5). The consumption of NADPH was immediately monitored at 340 nm for 20 min using the VersaMax microplate reader. The linear decrease in NADPH absorbance was used for calculating NADPH consumption and expressed as % of activity of drug-treated sample over that of DMSO-treated sample.

### Preparation of cell lysates and Western blot analysis

Cells were seeded onto 100 mm or 60 mm culture plates and upon reaching ~70% confluency, they were subjected to drug treatment for indicated durations. Pellets of compound-treated or DMSO control cells comprising both attached and floating cells were collected and lysed in lysis buffer (25 mM Tris-HCl pH 7.5, 100 mM NaCl, 2.5 mM EDTA, 2.5 mM EGTA, 20 mM NaF, 1 mM Na_3_VO_4_, 20 mM sodium β-glycerophosphate, 10 mM sodium pyrophosphate, 0.5% Triton X-100) with freshly added protease inhibitor cocktail (Roche Diagnostics). Prior to being used in Western blot analysis and/or cellular TrxR, GR, Trx and GPx activity determination, lysates were precleared by centrifugation and protein concentrations were determined using a modified Bradford assay (Bio-Rad Laboratories) as described in the manufacturer's manual. This procedure for collecting whole cell lysate was used for all experiments except for the Trx-redox state determination experiment. Equal amounts of protein (50 μg) in each lysate sample were separated by SDS-PAGE, followed by electroblotting onto nitrocellulose membranes. Electroblotted proteins on membranes were probed with a primary antibody and subsequently by a secondary antibody conjugated to HRP.

### Determination of TrxR and Trx activity in cell lysates by insulin reduction assay

Freshly collected cell lysates were used for measurement of activity of cellular Trx and TrxR. To measure TrxR activity, in each well of a 96-well plate, 25 μg of cell lysate was incubated in a final volume of 50 μl containing 85 mM Hepes (pH 7.6), 0.3 mM insulin, 10 μM rhTrx1, 2.5 mM EDTA and 660 μM NADPH for 40 min at 37°C. Controls containing lysates and all reaction reagents except rhTrx1 for each lysate sample were also set up. 200 μl of 1 mM DTNB in 6 M guanidine-HCl, 200 mM Tris-HCl pH 8.0 solution was added to quench the reaction. The amount of free thiols generated from insulin reduction was determined by DTNB reduction at 412 nm using the VersaMax microplate reader. To measure Trx activity, procedures were carried out similar to those for determining cellular TrxR activity, except that the cell lysates were incubated for 20 min with 300 nM recombinant rat TrxR in place of rhTrx1. Controls containing lysates and all reaction reagents except recombinant rat TrxR for each lysate sample were also set up. For each sample, Trx or TrxR activity was calculated as the absorbance at 412 nm subtracted from that of the corresponding control and expressed as a percentage of the activity measured in DMSO-treated cells.

### Determination of yeast GR activity and GR activity in cell lysates by glutathione reduction assay

To determine *in vitro* yeast GR activity, compounds of concentrations 1-100 μM were incubated with 20 nM yeast GR and 200 μM NADPH in a volume of 100 μl phosphate buffer (0.1 M sodium phosphate, 2 mM EDTA pH 7.5) for 1 h at room temperature. At the end of the incubation, 100 μl of 1 M GSSG (final concentration 500 μM) and 200 μM NADPH in phosphate buffer was added to each well. NADPH consumption was monitored by measurement of absorbance at 340 nm using the VersaMax microplate reader. The results were calculated based on decrease in absorbance in the initial 90 s and expressed as a percentage of the enzyme activity of the DMSO-treated sample. For determination of cellular GR activity, 25 μg of cell lysate was mixed with a solution of GSSG and NADPH in phosphate buffer to a final volume of 200 μl (final GSSG and NADPH concentrations 1 mM and 200 μM respectively). The enzyme activity was determined by measuring the decrease in absorbance at 340 nm for 10 min at 37°C and expressed as a percentage of the enzyme activity of that of the DMSO-treated sample.

### Determination of bovine GPx activity and GPx activity in cell lysates by GPx activity assays

For determination of *in vitro* bovine GPx activity, compounds of concentrations 1-100 μM were incubated with 100 nM bovine GPx, 20 nM yeast GR, 1 mM GSH and 200 μM NADPH in a volume of 100 μl phosphate buffer (0.1 M sodium phosphate, 2 mM EDTA pH 7.5) for 1 h at room temperature. H_2_O_2_ solution in phosphate buffer was added to initiate the reaction (final concentration 1.5 mM). NADPH consumption was monitored at 340 nm using the VersaMax microplate reader. The cellular GPx activity was determined similarly except lysates of HCT 116 and MCF-7 cells (25 μg protein) were used instead of bovine GPx. The results were calculated based on decrease in absorbance in the initial 3 min and presented as % of GPx activity of drug-treated sample over that of DMSO-treated sample.

### Determination of total cellular thiols

HCT 116 cells were seeded in 6-well plates and allowed to grow to 70-80% confluency. They were treated with compounds 4 and 5 (20, 30, 40 or 50 μM) for 4 h, after which collected lysates were used fresh for total cellular thiol determination. Briefly, in each well of a 96-well plate, 10 μl of each lysate sample was mixed with 90 μl of 1 mM DTNB in 6 M guanidine-HCl, 200 mM Tris-HCl pH 8.0. Absorbance readings measured at 412 nm were matched against a calibration plot obtained from a series of serially diluted GSH solutions (known concentrations in μM) in 50 mM Tris-HCl, 2 mM EDTA pH 7.5. The calculated total thiol content, normalized to per mg of proteins present in each lysate sample of compound-treated cells, was expressed as a percentage over that in DMSO-treated cells.

### Immunoprecipitation

HCT 116 cells were seeded in 60 mm plates and upon reaching 40% confluency, they were transfected with 1.5 μg of pCMV-SPORT6-ASK1 per plate. When the transfected cells reached ~70% confluency, they were treated with DMSO or compound 4 at indicated concentrations for 4 h and lysates were collected for immunoprecipitation set-up. For each lysate sample, a volume containing 1 mg of protein was mixed with 1.5 μg of anti-Trx antibody coupled to 20 μl of protein G-sepharose (GE Healthcare) and tumbled at 4°C for 2 h. The G-sepharose beads were washed three times in a cold washing buffer containing 20 mM Tris (pH 7.5), 0.5 M NaCl and 1 mM EGTA and once in a buffer containing 50 mM Tris (pH 7.5). The immunoprecipitated proteins were denatured in SDS sample buffer containing 5% β-mercaptoethanol and subjected to SDS-PAGE and Western blotting.

### Determination of Trx redox state in cells

The redox state of cellular Trx was assessed as previously described [42]. HCT 116 cells were seeded in 60 mm plates and allowed to grow to 70% confluency. Compounds 4 or 5 were then added to the cells for 4, 8 or 14 h. Lysates were collected in lysis buffer (50 mM Tris-HCl pH 8.3, 2 mM EDTA, 6 M guanidine HCl, 0.5% Triton-X, 50 mM IAA). The lysates were incubated in the dark for 30 min at room temperature, sonicated briefly, and finally passed through a MicroSpin G-25 column (GE Healthcare) to remove guanidine and unreacted IAA. The desalted lysates were subjected to native PAGE, followed by transfer of separated proteins onto nitrocellulose membranes for Western blot analysis using anti-Trx antibody.

### ROS determination

Oxidative stress was assessed through estimating the level of ROS using the H_2_DCFDA assay [58] with modifications. Briefly, HCT 116 cells were seeded in 6-well plates and incubated for 24-48 h at 37°C till 70-80% confluency. They were treated with H_2_O_2_ (200 μM) or compounds 4 and 5 (20, 30, 40 or 50 μM) for 3 h. Following treatment, cells were washed with PBS and incubated with 10 μM carboxy-H_2_DCFDA in PBS at 37°C in the dark for 45 min to allow reaction between taken up H_2_DCF and ROS. The cells were collected, washed with cold PBS, pelleted, resuspended in 200 μl of PBS and transferred to wells in a 96-well black fluorescence plate. The fluorescence intensities were measured at an excitation wavelength of 485 nm and an emission wavelength of 530 nm using a Tecan Infinite M200 Pro microplate reader. The results were expressed as a percentage of fluorescence intensities measured in drug-treated cells over those produced in DMSO-treated cells.

### BIAM labeling assay

To assess whether the Sec residue in mammalian TrxR was susceptible to indolin-2-one compounds, biotin labeling through BIAM alkylation of the free selenol at pH 6.5 in recombinant rat TrxR was performed using a previously described method with modifications [46]. A 100 μM BIAM stock solution in 100 mM Tris-HCl, 1 mM EDTA, pH 6.5 was prepared fresh for each experiment. NADPH-reduced recombinant rat TrxR (0.9 μM) was incubated with compound 4 or 5 (20 μM) or DMSO vehicle in 50 mM Tris-HCl, 1 mM EDTA, pH 7.5, and 200 μM NADPH at 37°C. At indicated timepoints, a 3 μl aliquot was withdrawn and mixed with 20 μM BIAM solution in 100 mM Tris-HCl, 1 mM EDTA, pH 6.5 for 15 min at 37°C to allow selective alkylation of free Sec residues. The reaction was quenched by adding freshly prepared IAA solution (final concentration 50 mM). The samples were then denatured in SDS sample buffer, boiled and subjected to SDS-PAGE and Western blotting. The biotin was detected by HRP-conjugated streptavidin using the enhanced chemiluminescence system. Membranes were stripped with stripping buffer (0.15 M glycine, pH 2.5, 0.4% SDS) and reprobed with polyclonal anti-rat TrxR1 antibody purified from rabbit antiserum against rat liver TrxR1 [59]. At each timepoint, 15 μl of compound-treated TrxR mixture was also removed for TrxR activity determination using the DTNB assay.

### Statistical and densitometric analysis

Numerical data are presented as means ± SD of different determinations. Student's *t*-test analysis was performed to compare means between control and treatment groups. ImageJ software (NIH) was used for quantification of intensities of Western blot bands.

## Abbreviations

ASK1: apoptosis signal-regulating kinase 1
DCF: dichlorodihydrofluorescein
GSH: glutathione
GSSG: glutathione disulfide
GPx: glutathione peroxidase
GR: glutathione reductase
JNK: c-Jun N-terminal kinase
Keap1: Kelch-like ECH-associated protein 1
MAPK: mitogen-activated protein kinase
NQO1: NAD(P)H:quinone oxidoreductase 1
Nrf2: nuclear factor erythroid 2 p45-related factor 2
PARP: poly(ADP)-ribose polymerase
ROS: reactive oxygen species
Sec: selenocysteine
SCA: supercinnamaldehyde
Trx: thioredoxin
TrxR: thioredoxin reductase
